# Multidecadal fluctuations in green turtle hatchling production related to climate variability

**DOI:** 10.1038/s41598-023-28574-4

**Published:** 2023-01-27

**Authors:** Pablo del Monte-Luna, Miguel Nakamura, Vicente Guzmán-Hernández, Eduardo Cuevas, Melania C. López-Castro, Francisco Arreguín-Sánchez

**Affiliations:** 1 Departamento de Pesquerías y Biología Marina, Instituto Politécnico Nacional, 23096 La Paz, Baja California Sur Mexico; 2 Departamento de Probabilidad y Estadística, Centro de Investigación en Matemáticas, 36023 Guanajuato, Guanajuato Mexico; 3 Área de Protección de Flora y Fauna Laguna de Términos, Comisión Nacional de Áreas Naturales Protegidas, 24140 Ciudad del Carmen, Campeche Mexico; 4 Recursos del Mar, CONACYT-Centro de Investigación y de Estudios Avanzados del Instituto Politécnico Nacional, 97310 Mérida, Yucatán Mexico; 5 Programa para la Conservación de Tortugas Marinas, Pronatura Península de Yucatán, A.C., 97205 Mérida, Yucatán Mexico

**Keywords:** Ecology, Population dynamics

## Abstract

The state of Campeche, Mexico, harbors one of the largest green turtle (*Chelonia mydas*) rookeries of the Wider Caribbean Region. Since the 1970s, harvesting of this population was common practice, but it has since ceased, and the population is rebounding as a consequence. In this rookery, during the past 37 years (1984–2020), the positive relationship between the annual number of nesting females and the number of hatchlings they produce has revealed a long-term population signal that we postulate could be related to environmental factors. To investigate this relationship more deeply, we adopt a stock-recruitment (SR) approach, which is commonly used in fisheries. Regression analysis methods for the SR relationship, including a dynamic version of the model that incorporates the effect of sea surface temperature, show that the number of recruits produced and the number of hatchlings per unit nester were significantly and inversely correlated with a 26-year cycle of the Atlantic Multidecadal Oscillation (AMO) with a three year lag. A possible explanation for this finding is that environmental conditions during warming periods of the 26-year AMO cycle may negatively affect hatchling production by altering the nest moisture content during the incubation period, and increasing embryonic mortality, while the annual female abundance at nesting beaches may decrease due to trophic effects. The time series of abundance corresponding to other population units of green turtles as well as other species of sea turtles in the Gulf of Mexico present a similar behavior to that evaluated here, suggesting a basin-wide environmental effect.

## Introduction

The green turtle (*Chelonia mydas*) has a circumglobal distribution throughout tropical and subtropical waters of the Atlantic, Indian and Pacific Oceans. The largest rookeries of the Wider Caribbean Region are located in the southwestern Gulf of Mexico^1^. Here, the green turtle populations nesting along the Campeche coast increased almost 2.5 orders of magnitude during the period 1984–2017^2^. Moreover, since the early 2000s, the annual number of hatchlings in other smaller population units that nest in different parts of the gulf (Tamaulipas, Veracruz, Quintana Roo, Yucatán, and Cuba) follows a similar trend to that observed in Campeche^3^ which is the most important rookery in the Yucatán Peninsula. Despite being a strategic region for chelonid conservation, the ecology of long-term population trends of sea turtles on the southeastern coast of Mexico has rarely been explored.

**Figure 1:**
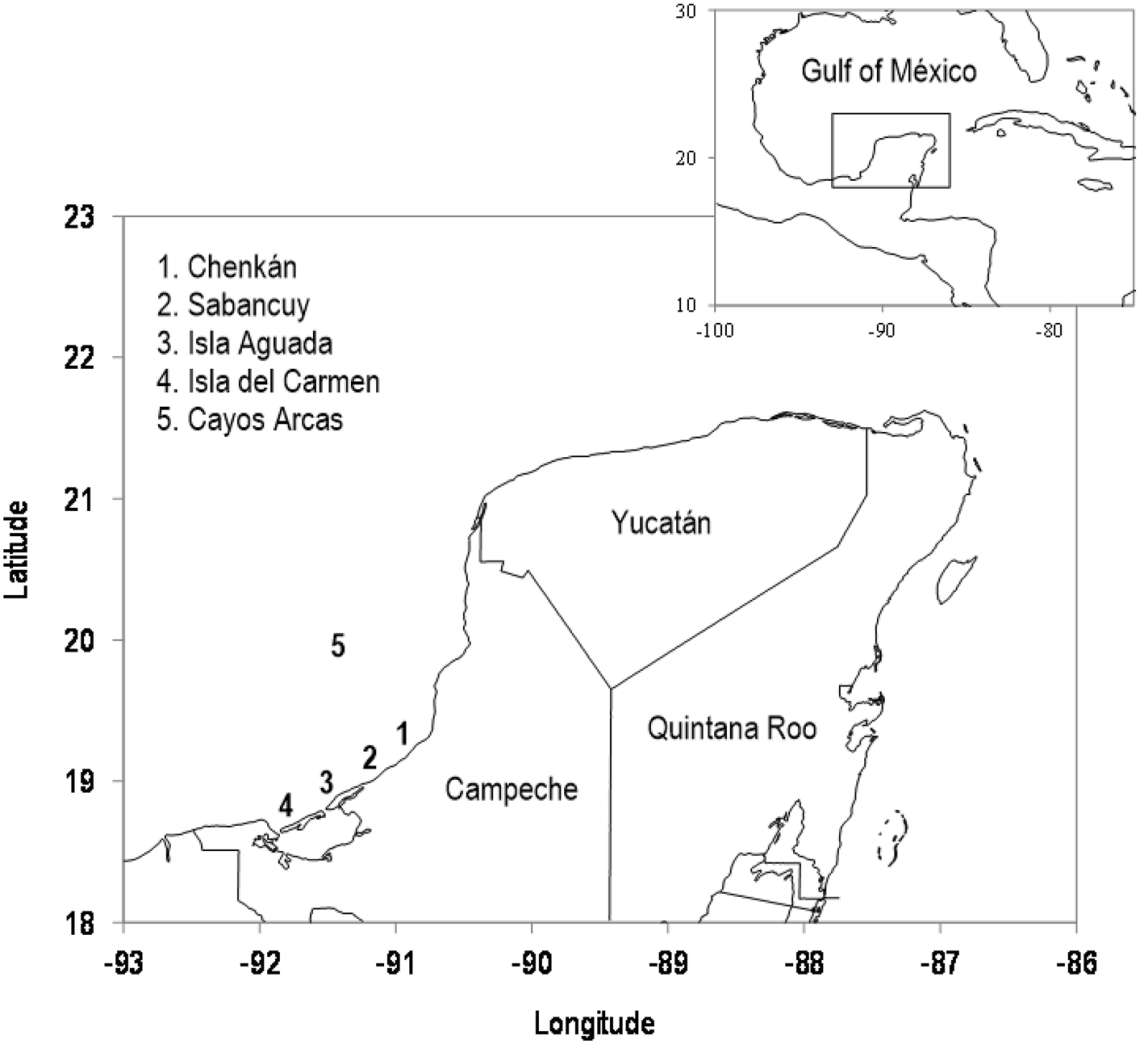
Study area showing the five main nesting beaches of the green turtle (*Chelonia mydas*) along the Campeche coast in the southwestern Gulf of Mexico. The map was generated in “Microsoft Excel 2016 MSO (version 2202 compilation 16.0.14931.20216) 32 bits”, as an XY scatterplot using longitude and latitude data.

In the late 1970s, after the green turtle population in Campeche was severely depleted by fishing, a 30-year recovery plan (Fig. 1) began with the implementation of the first chelonid conservation initiatives by the Mexican Federal Government and is still in place today^4,5^. The primary goals of these efforts are to monitor and increase the number of sea turtle hatchlings with the expectation that a fraction of them will eventually be incorporated into the nesting stock^6^. One product of such a long-term conservation process is a 37-year historical record of the annual number of green turtle nesting females and hatchlings, which is the basis of the study.

In sea turtle research, it is rare to encounter a continuous multidecadal time series of abundance^3,7,8^, and such dataset constitutes valuable information for demographic analyses of this group^9^. Coincidentally, the parent-progeny or stock-recruitment (SR) relationship is an analytical approach used for the population dynamics of marine organisms and is solely based on the time series of the annual abundance of adults and their offspring. This relationship describes how the nonreproductive fraction of a population (recruitment) is determined by the abundance of adults (stock)^10^; for instance, it can be used for estimating the recruitment production rate and the number of recruits per unit adult, as the abundance of adults changes. This approach was adopted for use in fisheries (marine fish and invertebrates). However, there are minimal applications of this approach for long-lived species such as sea turtles^11^.

The SR relationship has been modeled using a variety of curves, each presenting a different set of conditions. For example, in the Beverton–Holt function^12^ it is assumed that recruitment is constant at high levels of adult abundance, while in the Ricker equation^13^, it is assumed that recruitment is maximized at intermediate levels of adult abundance, thereafter decreasing asymptotically. Another stock-recruitment model is the power function proposed by Cushing^14^, where recruitment increases unrestricted as the abundance of the adult stock increases, but the recruitment rate can decrease. The premises of the first two models are that when a fishery begins, the population is near its carrying capacity and the stock is progressively reduced as the biomass extracted by fishing cannot be compensated by the population replacement rate over time. On the other hand, the Cushing model was originally conceived for representing the relationship between adults and recruits of recovering populations in the absence of fishing without reaching a plateau or carrying capacity.

**Figure 2:**
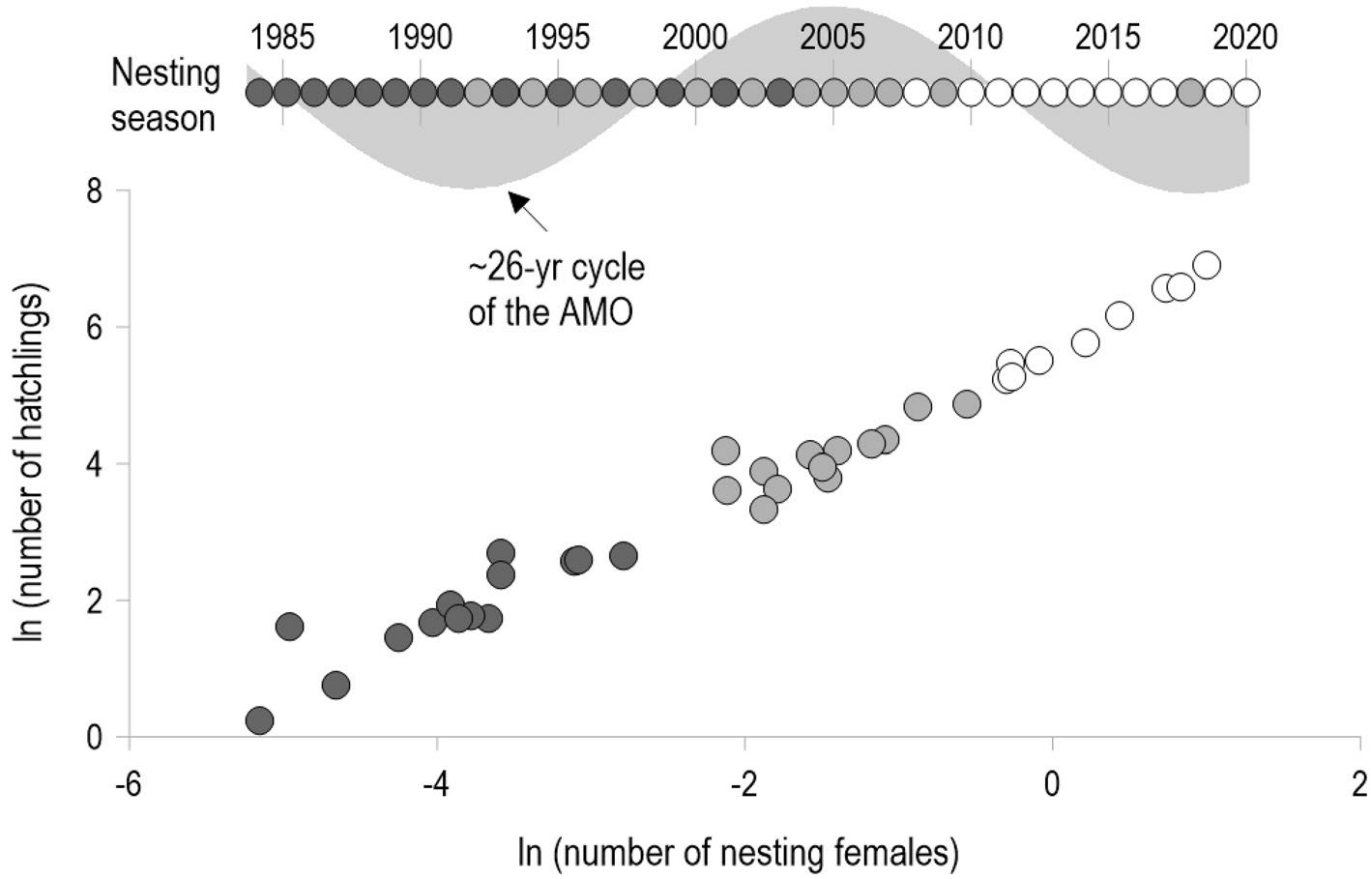
Relationship between the (ln) number of nesters and (ln) the number of hatchlings of the green turtle (*Chelonia mydas*) from Campeche in the southern Gulf of Mexico. The scatterplot shows three blocks of points discriminated by clustering analysis (*K*-means test)^3^. The time scale in the upper part of the graph represents the study period 1984–2020, and the circles are the positions of the elements of each block (differentiated by filling patterns) with respect to the calendar years. Behind the timeline in the upper part of the graph, depicted as a light gray area, a 26-year cyclic component of the Atlantic Multidecadal Oscillation Index (AMO, sea surface temperature over the Atlantic Ocean in the Northern Hemisphere) is underlined.

**Figure 3:**
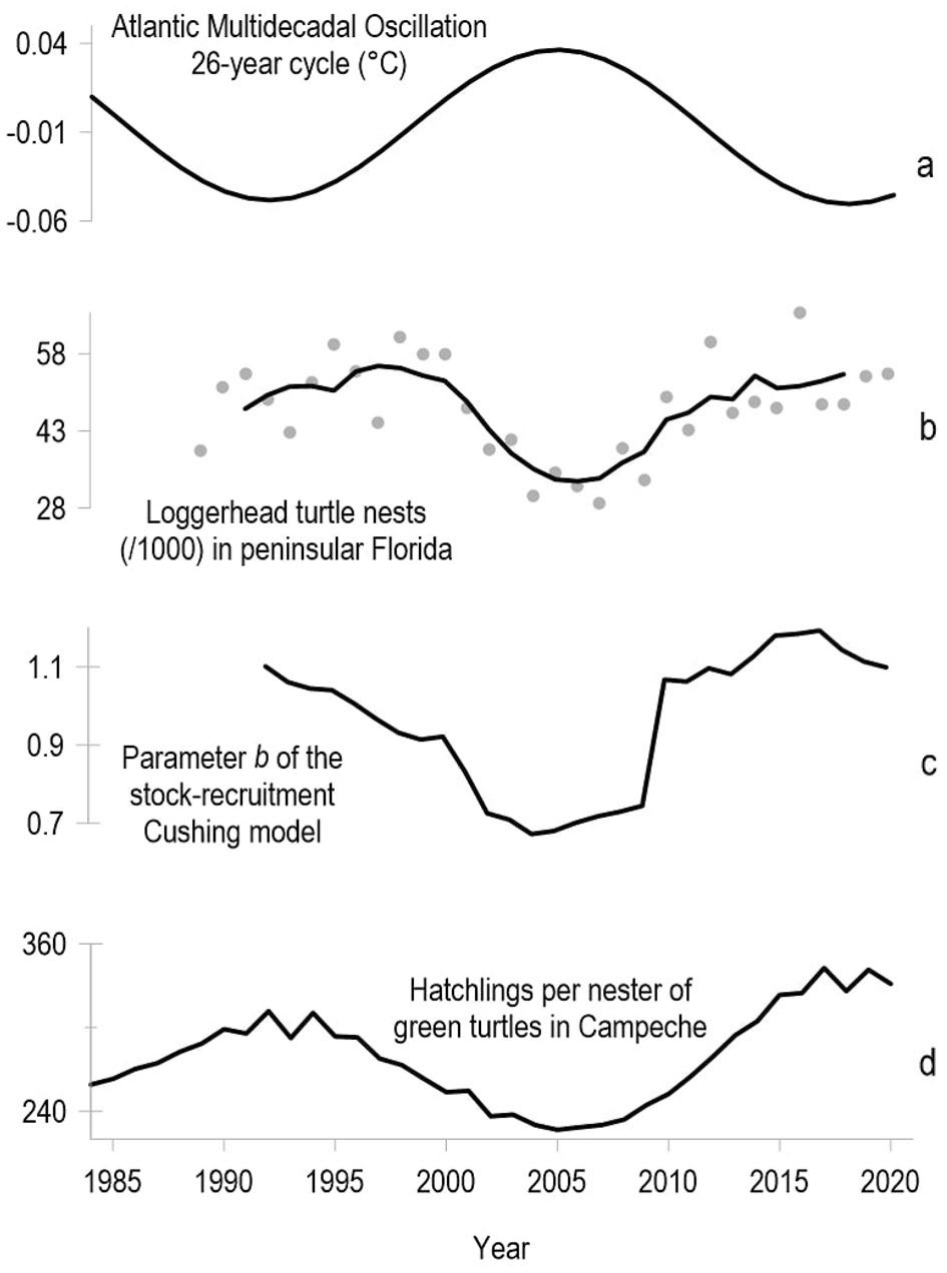
From top to bottom: (**a**) time series of a 26-year cyclic component of the Atlantic Multidecadal Oscillation Index (AMO, an index of sea surface temperature over the Atlantic Ocean in the Northern Hemisphere); (**b**) raw data (gray dots) and smoothed version (black line) of loggerhead turtle nests (/1000) in peninsular Florida; (**c**) parameter *b* of the Cushing stock-recruitment model (indicative of the hatchling production regime), fitted by local regression (9-year windows) using hatchling and nester abundance data 1984–2020 of the green turtle (*Chelonia mydas*) population from Campeche, Mexico; (**d**) the estimated recruitment rate for the green turtle (number of hatchlings per nesting female, Eq. (4)) derived from the extended Cushing model. The time series of parameter *b* from local regressions span from 1992 to 2020 because the estimates of each regression window are associated with the right endpoint (e.g., window 1984–1992 is assigned to 1992).

The premises of the Beverton-Holt and Ricker SR models can be illustrated by labeling each data point in a typical SR relationship (adult abundance is *X* and recruitment is *Y*) with the corresponding calendar years: the progression in time tends to occur from right to left as fishing reduces the adult stock. On the other hand, the premise of the Cushing SR model is that when a population has been depleted and is in the process of recovering under a regime of negligible fishing mortality, it begins to grow from very low numbers toward its carrying capacity^15^. In this scenario, the SR data are confined around small or intermediate values of stock abundance and the time progression of the data tends to occur from left to right, contrary to the way in which a typical SR relationship is interpreted. Knowing that the green turtle population in Campeche is recovering in the absence of fishing (or moderate fishing), we consider the Cushing SR model to be appropriate candidate for the present analysis.

Once an ad hoc SR model for representing key features of the green turtle population in Campeche is resolved, valid measures for stock and recruitment must be adopted. Recruitment is defined^16^ as the individuals that are alive at any specified time after the egg stage, and there are two measurement approaches: (I) take measurements close to the egg stage to clearly observe the relationship between eggs deposited and juvenile fish produced; and (II) take measurements based on the individuals recruited to the fishery to explore the relationship between the residual stock after fishing and the number of recruits at a later time. We chose the former measurement approach because fishing mortality is negligible in this case, and the assumptions for approach II are rather difficult to comply with given the available data. Thus, we assumed that hatchling numbers could constitute recruitment, which coincides with the definition in other studies^15,17^, and the abundance of nesting females constitutes the adult stock.

In a previous study^3^ it was shown that there are three chronologically ordered groups in the relationship between the abundance of nesting females and hatchling numbers of green turtles in Campeche during 1984–2020. Each group, 1984–1997, 1998–2009, and 2010–2020, contains between 10 and 13 observations (Fig. 2). The sequence and spans of these three regimes appear to agree with three distinct stages of a 26-year cycle of the Atlantic Multidecadal Oscillation (AMO, sea surface temperature—SST—over the Atlantic Ocean in the Northern Hemisphere). This particular cycle is identified as one of the dominant harmonic components of the AMO during 1900–2010^18^.

**Figure 4:**
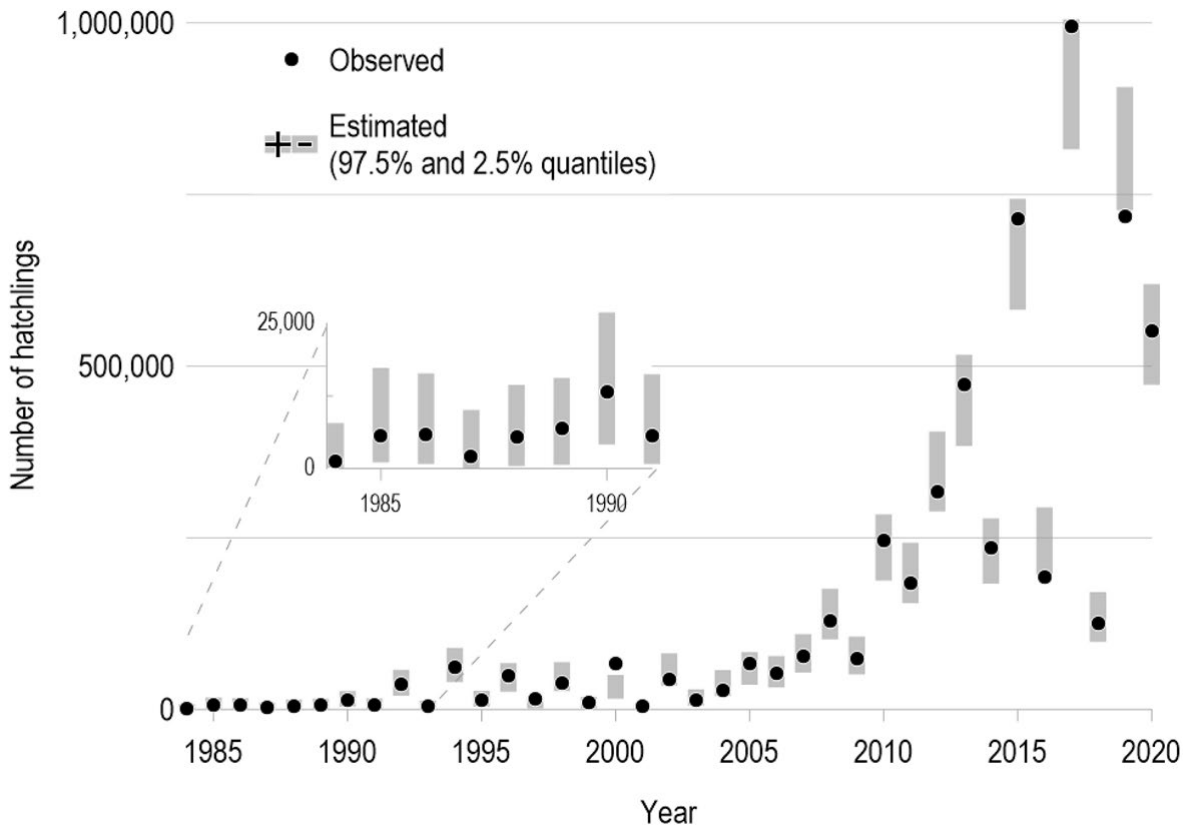
Observed number of hatchlings per year (*R*) of *Chelonia mydas* (black dots), with 95% prediction intervals constructed from fitting the extended Cushing model (Eq. (2)). The shaded regions outline 97.5% and 2.5% estimated quantiles of the positively-skewed distribution associated with hatchlings for a given sea surface temperature (from a 26-year cycle of the Atlantic Multidecadal Oscillation Index) and nesting females (*S*) for each year. This display shows that hatchling’s variance increases as its median (or mean) increases. The inset shows how the model fits the smallest values of the dataset.

We consider that a long-term pattern of variation is implicit in the SR relationship of the green turtles in Campeche and, furthermore, that a relationship with the AMO can be revealed by using the Cushing model. The relationship between sea turtle population dynamics and the AMO has been described for loggerhead turtles (*Caretta caretta*) in Florida^19^ and hawksbill sea turtles in the Yucatán Peninsula^18^. Based on the actual AMO behavior during the period of study (Fig. 2), we conclude that a relation between SR regimes and the AMO could be established if we observe the following pattern: the first regime is similar to the third, and both regimes are different from the second. We do not claim that a periodic signal in the green turtle population would be entirely proven with this finding, as the sampling range is only 37 years; we only assert that differences evidenced in the data synchronize with the limited portion of the AMO periodic behavior that matches the sampled dates.

The Cushing SR function has the form $R = a S^b$, where *R* is the number of hatchlings and *S* is the number of nesting females. Parameter *a* holds no biological meaning; it is merely a conversion factor to the units in which *R* is measured. However, the parameter *b* is a shape parameter, a crucial indicator of the type of regimen that *R* holds with respect to *S*: if $b > 1$, *R* increases “exponentially”; if $b = 1$, *R* increases linearly; and when $b < 1$, *R* increases “logarithmically”. Hatchling production is unrestricted by the abundance of nesting females in any case. However, when $b > 1$, the rate of hatchling production increases as the nester abundance increases, and diminishes when $b < 1$. If distinct, time-related clusters identified in the SR relationship of the green turtle population in Campeche^3^ correspond to different SR regimes, then it may be postulated that parameter *b* is also related to time. The novel contribution of this study is that the Cushing SR model was applied to test for the association between hatchling production and environmental parameters, specifically SST associated with the 26-year AMO cycle. The application of this model allows predictions of recruitment rate, and overall rookery production, factors that are very likely to be influenced by climate change.

The goals of the present study were (1) to determine if the pattern of variation of the rate of change of hatchlings (*R*) with respect to nesters (*S*) in the SR relationship of the green turtle in Campeche is correlated to a 26-year cycle of the AMO by tracking changes in Cushing’s *b* parameter; and (2) to build a dynamic Cushing SR model by means of a formal statistical formulation that explicitly incorporates this environmental variable for characterizing the distribution of the annual hatchling abundance of green turtles in Campeche for the period 1984–2020.

## Methodology

### Sea turtle monitoring and data acquisition

The historic hatchling and nester abundance data used in the present analysis (available in the Supplementary Information) represent the data collected from five sea turtle conservation camps installed and managed by distinct stakeholders in Campeche: Isla Aguada and Chenkan by the Comisión Nacional de Áreas Naturales Protegidas, Sabancuy by the Autonomous University of Carmen (UNACAR), Isla del Carmen by DECC, A.C. and GROSS, S.C., and Cayo Arcas by the Mexican Navy. For this study, a 100 km-long nesting beach on the coast of Campeche (Fig. 1) was monitored regularly at least three times per week during the nesting season (May-October) from 1984 to 2020. For monitoring, level 1 of protocol B, as defined by the Scientific Advisory Board of the State of the World’s Sea Turtles^20^ in their Minimum Data Standards for Nesting Beach Monitoring, was followed. Standard demographic and reproductive indicators such as the number of nests, eggs and hatchlings, hatchling success and number of nesting females were systematically recorded following the methodological specifications mandated by the official Mexican regulation NOM-162-SEMARNAT-2012 (effective from 2013 onward), in which the specifications for protecting, recovering and managing sea turtle populations in their nesting habitats are established.

From 1984 to 2012, the annual abundance of green turtle hatchlings in Campeche was derived from direct tallying (census) of the total number of nests, since all of them were relocated, so the error associated with counting was negligible^6^. From 2013 onward, two procedural adjustments took place. First, although the complete registration of nests in the season was maintained, the new regulation allowed relocating only those nests at imminent risk^6,21^, which represent approximately 10% of the total number of nests^6^. Second, in 2013, the number of nesting females and hatchlings coincidentally and dramatically increased, so a census became infeasible. A sample size of 25% nests was found to be representative of the total number of nests and hatchlings in the season to achieve 95% confidence^6,22^. Since 1984, the number of specialized technicians involved and their equipment have been directed to facilitate the personnel’s task in the field, maintaining the monitoring efficiency (constant number of beach surveys) and, from 2013 onward, ensuring that at least 25% of the total number of nests are surveyed each season^4,5^.

Similarly, from 2013 onward, 70% of the total number of nesting females (namely, the stock) were directly observed nesting at the beach and the remaining 30% were estimated using the number of nests not associated with direct female observation and the internesting intervals obtained from mark-recapture data^23^. In Campeche, the mean number of nests per individual during a single season is $3.98 \pm 0.12$ (range 3.4–4.2^23,24^); we rounded this number and assumed 4 nests per year per individual, which is consistent with other studies in the Yucatán Peninsula^25^. Moreover, for a collection of five turtle species, an error of 20% in the estimated annual abundance of nesting females is associated with sampling regimes with approximately 5% survey coverage^26^. In Campeche, the sampling coverage of green turtle nesters was more than 70% during the entire study period, so the sampling error for *S* was deemed to be considerably less than 20%.

### Smoothing technique: local regression

To investigate whether parameter *b* of the Cushing function describes a nonconstant, weaving behavior similar to the 26-year AMO cycle, the model was dynamically fitted to the green turtle SR data by successively sliding a 9-year window over the entire 1984–2020 span. Only data contained within a given window were used to estimate parameters *a* and *b*, which is a form of local regression^27^. The estimates of *a* and *b* were obtained using ordinary least squares, since the Cushing function permits the linearization $\ln (R) = \ln (a) + b \ln (S)$. Using the first nine data pairs (1984–1992) estimates of *a*, *b* associated with 1992 were produced. Estimates produced by the next window (1985–1993) were associated with 1993. This procedure was repeated until the last nine data pairs (2012–2020) were processed, resulting in a time series of estimated parameter values over 1992–2020. The 9-year window is arbitrary and was chosen as a compromise between creating too small a number of total points in the time series and relying on too few data points in any given window to produce meaningful estimates. The resulting time series of *b* parameters was compared to the 26-year AMO cycle.

### Statistical modeling: transformation-based regression

To rigorously test whether there is a Cushing relationship with a time-varying exponent, we formulated an explicit parametric statistical model that builds upon the Cushing SR function and allows for time variation in *b* through a proxy for the environment. Let $T_t$ denote the value of the temperature anomaly represented by the 26-year AMO cycle with a 3-year lag at year *t*. We first posit a simple linear relationship of the form

1 $$\begin{aligned} b_t = \gamma _0 + \gamma _1 T_t \end{aligned}$$

to account for the change in $b_t$ as a function of $T_t$. Then a regression model is superimposed to relate $S_t$ and $b_t$ to $R_t$. Parameter *a* is considered constant, albeit unknown, throughout. Understood as a statistical, nonlinear relationship between a response ($R_t$) and explanatory variables ($S_t$ and $T_t$), the conceived model, henceforth termed the extended Cushing model, is succinctly stated as follows:

2 $$\begin{aligned} R_t = \left( \sqrt{a S_t^{\gamma _0 + \gamma _1 T_t}} + \frac{\sigma }{2}\varepsilon _t \right) ^2, \end{aligned}$$

where $\varepsilon _t$ are non-observable, independent and standard normal perturbations representing variability occurring haphazardly in nature. Parameters *a*, $\gamma _0$, $\gamma _1$ and $\sigma > 0$ are unknown parameters to be estimated from data that characterize the statistical relationship between the response and explanatory variables via the Cushing relationship.

Abundant details regarding this distinctive model, its rationale, its main properties, maximum likelihood estimation of parameters, and results pertaining to its empirical validation are provided in the Supplementary Information. Relevant facts are highlighted here for clarity to ensure the proper interpretation of what follows.

If $\varepsilon _t = 0$ (no perturbation), or equivalently, if $\sigma \approx 0$, the model reproduces the Cushing relationship exactly, so that this SR model is embedded and honored. Larger values of $\sigma$ signify that observed data wander away randomly from the relationship to a greater degree.

Whereas $\sigma$ is conceived to account for variation, it is parameters *a*, $\gamma _0$ and $\gamma _1$ that possess main biological interest. Namely, the median value of *R* for given *S* and *T* is no less than the Cushing model itself,

3 $$\begin{aligned} \text {med}(R) = a S^{\gamma _0 + \gamma _1 T}. \end{aligned}$$

An interpretation of the regression model with respect to an observation of *R* follows: the Cushing SR relationship is randomly perturbed upward or downward (asymmetrically, because of the square root) depending on the sign of $\varepsilon$. Furthermore, the possibility that *T* bears no association at all with *b* or with *R* is accounted for, since $\gamma _1 = 0$ is allowed.

The recruitment rate (number of hatchlings per unit nester, RR), obtained by differentiating $\text {med}(R)$ as a function of *S*, is

4 $$\begin{aligned} \text {RR} = a \left\{ \gamma _0 + \gamma _1 T \right\} S^{\gamma _0 + \gamma _1 T - 1 }. \end{aligned}$$

Estimates of *b*, $\text {med}(R)$ and RR functions of *S* and *T* are derived by plugging individual parameter estimates (Table 1) into (1), (3), and (4).

Figures in main text were generated in Grapher 16.0.314 (64-bit) and all statistical computations were achieved in R^28^ using standard non-linear least-squares functions. R code for parameter estimation and diagnostic procedures used for model (2) may be found at https://github.com/mnakamuramx/Chelonia.

## Results

Regarding smoothing for preliminary exploratory purposes, the estimated values of parameter *b* that resulted from applying the Cushing model as local regressions to the green turtle SR data from Campeche, supported the notion that *b* is not constant (Fig. 3c and Fig. S1 in the Supplementary Information) and revealed a long-term population signal with a swaying drift and a return period of approximately 25 years, which is similar to the (smoothed) time series of the population indices of loggerhead turtles in Florida^29^ (Fig. 3b). The time series of parameter *b* was compared with lagged versions of the 26-year cycle of the AMO and found to be inversely and maximally correlated when the lag was fixed at 3-year ($r^2 = 0.83$; $p < 2.2\text {e}{-16}$).

On the other hand, the extended Cushing model, (2), fit the data well; the correlation between the observed and estimated values of *R* was $r^2 = 0.98$, $p < 2.2\text {e}{-16}$ (Fig. 4). Moreover, the probabilistic structure in (2) was realistic in regard to assumptions of normality and independence (see Supplementary Information). The estimated regression parameters by the maximum likelihood were all significantly non-zero (Table 1); the estimate for parameter $\sigma$ is 50.65, consistent with the fact that observations display noticeable variability. The time series of RR values (Fig. 3d), estimated from Equation (4), was also negatively correlated with the SST at a 3-year lag ($r^2 = 0.89$, $p < 2.2\text {e}{-16}$). The RR value during the maximum of the AMO cycle (warmer SST conditions) in 2005 was 227, while during the last minimum of the cycle in 2017 (colder SST conditions) the RR was 227 hatchlings per nester as a function of *S* (Fig. 3d).

There are two characteristics apparent in the data in their original scale for *S* and *R*: heterogeneous variation (i.e., increased variance in *R* as its median value increased) and positive skewness (i.e., asymmetry in the sense that extreme high-ended values occur with greater probability than low-ended ones). The square root transformation employed in the model’s stochastic structure (2) implicitly accounted for both of these features and was reflected in the calculated prediction intervals (Fig. 4 and Supplementary Information). The fact that the estimate for $\gamma _1$ is significantly non-zero has an important implication: a change in *T* is associated with a change in the distribution of *R*, characterized by the relationship given by (2).

**Table 1:** Maximum likelihood estimated parameters, 95% confidence intervals endpoints, and descriptive statistics of the extended Cushing stock-recruitment model (2) applied to demographic data of the green turtle population nesting in Campeche, Mexico.

Parameter	Estimate	StdErr	2.50%	97.50%	*t*-value	$\Pr (>|t|)$
*a*	264.299	53.382	171.670	395.437	4.951	$1.99\text {e}{-05}$
$\gamma _0$	0.999	0.032	0.936	1.066	31.531	$<2\text {e}{-16}$
$\gamma _1$	$-\,0.615$	0.167	$-\,0.955$	$-\,0.278$	$-\,3.692$	0.000776

The model inputs were the number of nesting females (*S*) and the number of hatchlings (*R*). The parameters of the extended Cushing model incorporated an association with the 26-year cycle of the Atlantic Multidecadal Oscillation.

## Discussion

Here we extended the use of the Cushing SR model from fishery assessments to the population dynamics of the green turtles assuming that the nesting females were representative of the adult stock and the hatchlings were the recruits. The extended Cushing model is a generalization akin to those of other studies^17,30^ in regard to incorporating environmental variables that alter the SR relationship. However, this was achieved here by allowing an individual parameter in an SR model to directly depend on the covariates instead of devising a certain mathematical perturbation term and then using it to modify the SR by addition^30^ or by multiplication^17^. Moreover, while previous studies^19,31^ correlated highly variable time series of demographic indicators with raw annual values of environmental surrogates, here we used a single 26-year periodic signal of the AMO for constructing a dynamic population model for green turtles. It must be noted that the interpretation of the parameters of the extended Cushing model and the derived quantities must be strictly circumscribed by the adult stock and recruitment as they were assumed and within their observed ranges. Neither the status of any other life stages, nor the behavior of this SR relationship outside those ranges should be inferred from the solutions developed.

### SR regimes, recruitment rate and the modeling of hatchling abundance

We acknowledge that the 3-year lag in the correlation between the 26-year AMO cycle, parameter *b* and the recruitment rate, indicates that SST is not causative. In fact, the lag in the correlation between the AMO and the sea turtle population time series seems to be a persistent but inexplicable feature^19,31,32^. If SST is not the direct cause, then green turtle hatchling production in Campeche seems to respond to a yet unidentified, large-scale environmental factor (or a suite of them) that changes with a similar periodicity to that of the 26-year harmonic component of the AMO. Therefore, beyond postulating an ecological mechanism, some regional environmental conditions with a potential influence on hatchling production and the recruitment rate of green turtles in Campeche that occur during Atlantic-wide cooling and warming episodes will be described.

During warmer years, the low soil humidity tends to be suboptimal for oviposition compared to colder years, and females 191 crawl for longer distances during the nesting season, perform more nesting attempts and spend more energy finding suitable nesting spots with higher humidity, which translates into fewer nests and fewer eggs per season 6, 32 192, i.e., lower recruitment rates. Additionally, in warmer years, lethal temperatures occur more frequently than in cooler years, causing higher embryonic mortality^33^ and, ultimately, lower hatchling production. Nesting green turtle females may also be affected by changing sea temperatures via trophic dynamics^34^. The green turtle, being a mostly herbivorous species, feeds on food sources (primary producers) that are more highly impacted by environmental change than other trophic levels. During warming periods, productivity in coastal ecosystems along the Yucatán Peninsula tends to decrease^35^, which may reduce feeding opportunities for sea turtles during preparation for breeding, later resulting in longer remigration intervals^36^ (time between nesting years) and fewer females that acquire sufficient energy for nesting^37,38^. Inverse correlations between SST and the number of nesting females, number of nests and clutch size have also been found for green turtles in the Pacific^39^ and Atlantic Oceans^40^ as well as in other species of sea turtles in the Yucatán Peninsula^32,41^.

The existence of long-term signals in sea turtle populations in the Gulf of Mexico is worth further investigation. For instance, population models of loggerhead turtles in Florida showed that environmental conditions represented by the AMO at the time of hatchling explain up to 70% of the annual nesting variability 30 years later^19^. This correlation could be a statistical artifact, since it remains significant at shorter lag times^31^; however, there is a clear multidecadal signal in the Florida Index Nesting Beach^29,42^, which is similar to that of parameter *b* in the SR relationship of the green turtle from Campeche (Fig. 3c) and to the 26-year cycle of the AMO. In either species, it remains unclear whether today’s nesting female abundance was influenced by cyclic environmental factors at the moment of hatching some decades ago, or if biological processes controlling female and hatchling turtles co-occur with those cyclic factors^43^.

The results of applying the extended Cushing model showed that the median of the probabilistic distribution of the annual number of green turtle hatchlings in Campeche was dependent on the abundance of nesting females and a low-frequency, basin-wide SST signal. It was not surprising that the annual number of nesters emerged as a steadfast predictor of hatchling production (Fig. 2), as has been demonstrated in other instances^9^. Notably, by means of a dynamic modeling framework, it was possible to unveil a multidecadal signal in basic population features that is implicit in a time series of demographic metrics showing an exponential trend and high interannual variation.

Moreover, the more unrealistic assumptions of fishery models previously applied to sea turtles^44^ were overcome. First, neophyte turtle abundance (recruitment to the reproductive stock) was not estimated from hatchling numbers several decades ago, as has been done in other instances^31,44^; instead, estimates were obtained by exclusively reframing an SR model as the relationship between nesters and hatchlings of the same year. Second, it was shown that parameter *b* of the Cushing model (and the recruitment rate) is closely related to a 26-year cycle of an environmental variable in an SR relationship of a population subjected to moderate to negligible fishing. Third, the SR model was enriched by setting its parameters as the functions of such a variable. We invite readers to explore these methodological guidelines as a first approximation for analyzing nester and hatchling abundance data of recovering sea turtle populations, but advise due caution regarding the technical and contextual assumptions indicated in the Supplementary Information.

### Relevance for green turtle conservation

The overall abundance trend of green turtle hatchlings and nesters in Campeche may reflect the effects of sustained conservation efforts. In Southeast Mexico, since the 1980s, improvements in logistics and human resources for surveiling and protecting sea turtles^5,23^ has resulted in an exponential increase in the pooled regional turtle abundance, which is roughly similar to the regional abundance trends of other population units of green turtles^4^. Currently, the number of green turtles in Campeche indicates that the regional nesting population is successfully recovering^3^, as are other populations of the species in other regions of the world^45–47^.

We believe that the results of the present study transcend the local scope for three reasons: (1) time series (2000–2020) of hatchling numbers from other parts of the Gulf of Mexico show a similar trend to those in Campeche; therefore, if the analytical approach undertaken here were to be applied using nester and hatchling abundance data from those nesting sites, similar results would likely be found. In other words, the different regimes in the SR relationship and the oscillating behavior over time of the Cushing model parameter *b* could be a common feature of green turtle populations across the entire gulf; (2) the existence of a similar signal to the one identified here in nest counts of loggerhead turtles in Florida is suggestive of a large-scale environmental effect on at least two species of sea turtles (pertaining to different trophic levels) within the Gulf of Mexico; and (3) if SST continues rising in the gulf, whether caused by the natural evolution of the 26-year cyclic component of the AMO^18^ (Fig. 3a) or by the positive trend in regional temperature fields attributable to anthropogenic climate change^48^, then a regime of low hatchling production and a reduction in the number of hatchlings per nester in the next decade is a possibility for this species in the Gulf of Mexico. Conservation efforts aimed at monitoring the nesting population and securing critical habitats for sea turtles along the Campeche coast are necessary for balancing a possible sustained decrease in hatchling production, especially if the warming trend in SST is concomitant with other stressors for sea turtles, such as sea level rise^48^.

Almost forty years have passed since the green turtle population nesting in Campeche numbered as few as six females; in 2017, the nesting population peaked at 2,728 nesting females. While these figures justify a certain degree of optimism regarding the recovery of regional population units of green turtles, by using a simple SR modeling procedure, we suggest that behind this positive trend in green turtle numbers, the hatchling production and recruitment rate is currently beginning to decrease as a response to environmental variability, whose long-term effects on the population are yet to be seen.

## Supplementary Information

Supplementary Information.

## Data Availability

All data generated or analysed during this study are included in its supplementary information files.
