# Author Correction: Multidecadal fluctuations in green turtle hatchling production related to climate variability

**DOI:** 10.1038/s41598-023-30419-z

**Published:** 2023-02-23

**Authors:** Pablo del Monte-Luna, Miguel Nakamura, Vicente Guzmán-Hernández, Eduardo Cuevas, Melania C. López-Castro, Francisco Arreguín-Sánchez

**Affiliations:** 1 Departamento de Pesquerías y Biología Marina, Instituto Politécnico Nacional, 23096 La Paz, Baja California Sur Mexico; 2 Departamento de Probabilidad y Estadística, Centro de Investigación en Matemáticas, 36023 Guanajuato, Guanajuato Mexico; 3 Área de Protección de Flora y Fauna Laguna de Términos, Comisión Nacional de Áreas Naturales Protegidas, 24140 Ciudad del Carmen, Campeche Mexico; 4 Recursos del Mar, CONACYT-Centro de Investigación y de Estudios Avanzados del Instituto Politécnico Nacional, 97310 Mérida, Yucatán Mexico; 5 Programa para la Conservación de Tortugas Marinas, Pronatura Península de Yucatán, A.C., 97205 Mérida, Yucatán Mexico

Correction to: *Scientific Reports* 10.1038/s41598-023-28574-4, published online 27 January 2023

In the original version of this Article, Miguel Nakamura was omitted as a corresponding author. Correspondence and requests for materials should also be addressed to nakamura@cimat.mx.

The original Article has been corrected.

