# Supplementary material for: Multidecadal fluctuations in green turtle hatchling production related to climate variability
Source: Sci Rep. 2023 Jan 27;13:1542. doi: 10.1038/s41598-023-28574-4 (PMC9883438; doi:10.1038/s41598-023-28574-4)
Supplement: Supplementary file 1 — Supplementary Information. [file 41598_2023_28574_MOESM1_ESM.pdf]

Multidecadal fluctuations in green turtle hatchling production related to climate variability (Supplementary Information)

Pablo del Monte-Luna[1,*], Miguel Nakamura[2], Vicente Guzmán-Hernández[3], Eduardo Cuevas[4], Melania C. López-Castro[5], and Francisco Arreguín-Sánchez[1]

[1]Departamento de Pesquerías y Biología Marina, Instituto Politécnico Nacional, 23096, La Paz, Baja California Sur, Mexico
[2]Departamento de Probabilidad y Estadística, Centro de Investigación en Matemáticas, 36023, Guanajuato, Guanajuato, Mexico
[3]Área de Protección de Flora y Fauna Laguna de Términos, Comisión Nacional de Áreas Naturales Protegidas, 24140, Ciudad del Carmen, Campeche, Mexico
[4]Recursos del Mar, CONACYT-Centro de Investigación y Estudios Avanzados del Instituto Politécnico Nacional, 97310, Mérida, Yucatán, Mexico
[5]Programa para la Conservación de Tortugas Marinas, Pronatura Península de Yucatán, A.C., 97205, Mérida, Yucatán, Mexico
[*]pdelmontel@ipn.mx

ABSTRACT

Technical details on the modeling approach that incorporates the Cushing stock-recruitment function into a regression model (the extended Cushing model) are discussed in regard to (a) the statistical formulation, (b) parameter estimation, (c) model diagnostics and validation, and (d) limitations and cautionary remarks.

Exploratory plots based on local regressions

In the main text, the preliminary analysis, which led to the working hypothesis of a relationship between b and SST based on local regression, was described. The Cushing model was dynamically fit by sliding a 9-year window over the entire 1988–2020 span of data from left to right (earlier to more recent). The corresponding confidence intervals (Fig. S1) for b are wide, as anticipated, because of the smaller sample sizes induced by the binning scheme, but the simple fact that some of them do not overlap supports the notion that b is not constant.

The modeling approach

The present context, where a theoretical parametric model $Y = f(X,\theta)$ to be considered preexists and is observed with random noise in practice, is very well-suited for an approach based on transformations[1,2]. In our stock-recruitment (SR) application, $Y = R$, $X = (S,T)$, $\theta = (a,\gamma_0,\gamma_1)$, and f is the Cushing relationship. This methodolody, termed the transform-both-sides (TBS) methodology, has been successfully applied for models arising in the SR realm[3]. The main idea is to transform the theoretical model so that the relationship between the variables it governs is left unaltered but allows for a vast array of different ways in which probabilistic disturbances can be introduced into the theoretical relationship.

Consider the Box-Cox family of power transformations, defined by

$$h_\lambda(y) = \begin{cases} \frac{y^\lambda - 1}{\lambda} & \text{if } \lambda \neq 0 \\ \ln(y) & \text{if } \lambda = 0. \end{cases} \tag{1}$$

It is assumed that for some power λ, applying this transformation prepares the theoretical model to be disrupted by an additive, normally-distributed random term:

$$h_\lambda(Y) = h_\lambda[f(X,\theta)] + \sigma\varepsilon, \tag{2}$$

where ε is a standard normal random variate and $\sigma > 0$. The value $\lambda = 1$ corresponds to "no transformation needed," giving rise to the common nonlinear model $Y = f(X,\theta) + \sigma\varepsilon$. A concave transformation (rendered by $\lambda < 1$) is expected because

R readily appears to be positively skewed, with its variance increasing with its mean. This is the reason why logarithmic transformation ($\lambda = 0 < 1$) is so conspicuous in applications, specifically in SR situations[4] and other circumstances in biology, chemistry and economics.

In fact, we initially experimented with $\lambda = 0$. The Cushing model becomes linear with a log transformation: $\ln(R) = \ln(a) + \gamma_0 \ln(S) + \gamma_1 \mathrm{T} \ln(S)$. Linear least squares could have easily been used for parameter estimation. However, we found that $\lambda = 1/2$ (a square root transformation, somewhat less severe than the logarithm transformation) was far more appropriate for the data. As shown in the next section, the reason for this choice is that the analysis of residuals revealed that the assumption of independent and normally distributed ε with constant variance is tenable. Applying the inverse $h_\lambda^{-1}(x) = (\lambda x + 1)^{1/\lambda}$, where $\lambda = 1/2$, to both sides of (2) yields the model postulated in the main text.

For given data, maximum likelihood estimation using the (2) model involves minimizing sums of squares[2] of quantities $h_\lambda(Y_t) - h_\lambda[f(X_t, \theta)]$ employing all available observations (R_t, S_t, T_t) (at once, with no grouping due to moving windows as described in the main text). This can be achieved with standard nonlinear least squares software to provide estimates for the model parameters in (2) as well as standard errors and confidence intervals.

An important byproduct of the statistical model presented here is an explicit distribution for Y given X. Regardless of the value of λ, the median of this distribution is always $f(X, \theta)$, yielding a convenient interpretation for the given theoretical model. This distribution is potentially useful for implementing simulation studies involving Y and for evaluating scenarios based on simulated, plausible or estimated values of X in future years. To simulate values of Y for given or estimated values of the parameters θ, σ, and λ, a standard normal variate ε is simulated. Then, compute

$$Y = \{\lambda h_\lambda[f(X, \theta) + \sigma\varepsilon] + 1\}^{1/\lambda}. \tag{3}$$

By considering quantiles, the distribution enables the construction of prediction intervals for Y for a given X and T, as shown in Fig. 4 of the main text. The p-th quantile for Y (that is, the number c such that $P(Y \leq c) = p$) is given by

$$q_p(Y) = \{\lambda h_\lambda[f(X, \theta) + \sigma z_p] + 1\}^{1/\lambda}, \tag{4}$$

where z_p is the p-th standard normal quantile (e.g., $z_{0.95} = 1.64$ and $z_{0.5} = 0$). Taking $p = 1/2$ and $\lambda = 1/2$ in this expression yields Equation (3) in the main text (the median). Probabilistic statements regarding Y also become accessible by knowing the distribution, e.g., the probability that Y will exceed (or fall short of) a given threshold of particular biological interest for the conservation of sea turtles.

Analysis of residuals

In the TBS model (2) where $\lambda = 1/2$, noncorrelated error terms that are normally distributed with equal variance are assumed. Figures S2, S3 and S4 are used as diagnostic tools to verify different aspects of these assumptions. All plots in the Supplementary Information were built using R[5] and ggplot2[6].

Remarks on the statistical model and important caveats

We must point out that the model described here is specifically for the data at hand and the specific context and should not be expected to hold unequivocally for all situations, e.g., for exploited populations or populations that are not in a recovery phase. Diagnostics must be checked for the distributional assumptions being made, as we have done. Functional relationships incorporating nonconstant parameters have been proposed, and there is also the danger of spurious relationships with the environment to be found[7]. However, the parameters of the stock-recruitment relationship themselves have been allowed to vary over time, either discretely in time (regime shifts) or as functions of covariates[8]. These approaches could, in principle, allow for more adaptive conservation actions that are responsive to climate change and other factors.

The fact that females input into the model are subject to measurement error is a legitimate source of concern[9]. This phenomenon is referred to as errors in variables in regression models[10] and gives rise to problems that may be severe when inferring model parameters, mainly estimation bias. We conducted a simulation study—detailed below—to investigate if errors with an order of magnitude of 10% in terms of numbers of females induce appreciable bias, and the answer is no. However, affirming errors in variables that are small is contingent on the context of the dataset, particularly at the data acquisition stage. Other contexts may be unsuitable for disregarding errors in variables.

Another problematic issue[9] is time series bias, which, in an exploited population, is the effect of the recruitment strength of a given year on the stock size at a later time, particularly when recruitment is measured based on individuals who are about to enter or who recently entered the spawning stock. Here, fishing and environmental processes do not operate for a sufficient amount of time to dampen the effect of the recruitment strength on adult abundance. In our study, the green turtle population

was subject to negligible fishing mortality, and there was at least a 20-year gap (age at first maturity of the green turtle[11]) between hatchlings and first-time nesting females, so the time series bias can be reasonably expected to be small.

As they may exert undue influence on estimated parameters, outlying observations have also been noted as problematic[9]. However, we did not identify outliers in our data (Fig. 4 in the main text). We observed heavy tails induced by a skewed distribution that gave rise to extreme values with high probability. Rather than labeling these extreme observations as outliers or rarities, we explain or accommodate these larger values via a statistical distribution. Thus, "outliers" are not deemed errors or abnormal realizations but simply manifestations of a physical phenomenon exhibiting a characteristic pattern of variation.

Simulation study for quantifying effects of errors in variables

To quantify the possible effects of errors in variables, we conducted a small simulation study. We repeatedly (10,000 times) generated artificial datasets of the same size as the original, using distributions derived from the original fit. However, the variable number of females was intentionally contaminated about its observed value; that is, errors in variables were introduced. We assumed a constant coefficient of variation (CV) and normally distributed contamination. For example, if CV $=$ 10%, and the number of females was observed to be 500, then the contaminated value was simulated as a normal variate with a mean of 500 and a standard deviation of $0.10 \times 500 = 50$ (larger estimates of the number of females are prone to larger variances). Parameter estimates were obtained by nonlinear least squares for each replicate, and histograms were constructed to show the sampling distribution of parameter estimates.

Here, we present sets of histograms (Fig. S5) for four illustrative cases: CV $=$ 0 (no errors-in-variables), CV $=$ 10% (small errors), CV $=$ 20% (moderate errors), and CV $=$ 40% (extreme errors). As previously mentioned, in our analysis, the error is most likely closer to 10% than to 20%. The red lines depict the true value of a parameter, and any concentration of parameter estimates centered (blue line) that is not on the red line represents bias. Bias is nonexistent when CV $=$ 0, as theoretically expected, and quite evident when CV $=$ 40%. Progressive increases in variance are also very apparent. However, bias is not severe when CV $=$ 10%, but it becomes compromised when CV $=$ 20%.

Data availability

The data used in the study are shown in Table S1.

References

1. Carroll, R. J. & Ruppert, D. Power transformations when fitting theoretical models to data. *J. Am. Stat. Assoc.* **79**, 321–328 (1984).
2. Carroll, R. J. & Ruppert, D. *Transformation and weighting in regression* (Chapman and Hall/CRC, 2017).
3. Ruppert, D., Cressie, N. & Carroll, R. J. A transformation/weighting model for estimating Michaelis-Menten parameters. *Biom. J. Int. Biom. Soc.* 637–656 (1989).
4. Iles, T. C. A review of stock-recruitment relationships with reference to flatfish populations. *Neth. J. Sea. Res.* **32**, 399–420 (1994).
5. R Core Team. *R: a language and environment for statistical computing*. R Foundation for Statistical Computing, Vienna, Austria (2021).
6. Wickham, H. *ggplot2: elegant graphics for data analysis* (Springer-Verlag New York, 2016).
7. Walters, C. J. & Collie, J. S. Is research on environmental factors useful to fisheries management? *Can. J. Fish. Aquatic Sci.* **45**, 1848–1854 (1988).
8. Sharma, R., Porch, C. E., Babcock, E. A., Maunder, M. N. & Punt, A. E. Recruitment: theory, estimation, and application in fishery stock assessment models. *Fish. Res.* **217**, 1–4 (2019).
9. Hilborn, R. & Walters, C. *Quantitative fisheries stock assessment: choice, dynamics and uncertainty* (Chapman & Hall, 1992).
10. Carroll, R. J., Ruppert, D., Stefanski, L. A. & Crainiceanu, C. M. *Measurement error in nonlinear models: a modern perspective* (Chapman and Hall/CRC, 2006).
11. Zug, G. R., Balazs, G. H. & Wetherall, J. A. Age and growth of Hawaiian green sea turtles (*Chelonia mydas*): an analysis based on skeletochronology. *Fish. Bull.* **100**, 117–127 (2002).

Year	Nesting females	Hatchlings	Year	Nesting females	Hatchlings
1984	6	1267	2004	153	28140
1985	26	5697	2005	247	66192
1986	23	5888	2006	224	52296
1987	10	2153	2007	337	77448
1988	18	5377	2008	574	128801
1989	20	6876	2009	308	73612
1990	45	13095	2010	920	246515
1991	21	5715	2011	742	184711
1992	121	36704	2012	1241	316224
1993	14	4251	2013	1540	473273
1994	207	62178	2014	763	235000
1995	46	13310	2015	2090	713872
1996	153	48556	2016	769	193385
1997	28	14667	2017	2728	995040
1998	167	37652	2018	418	124751
1999	28	10725	2019	2455	717783
2000	120	66424	2020	1685	551077
2001	7	5009			
2002	232	44196			
2003	62	14103			

Table S1. Annual number of nesting females and annual number of hatchlings of green turtles (*Chelonia mydas*) in Campeche, Mexico.

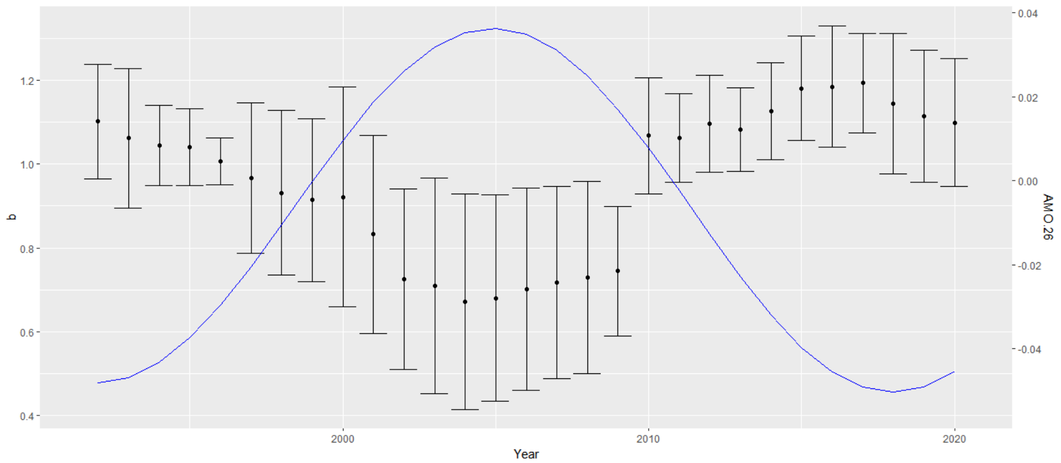

Figure S1. Estimated values of the *b* parameter in the Cushing relationship using local regression windows described in the main text. The intervals represent 95% confidence intervals for the estimates. Superimposed, in blue, is 26-yr AMO cycle.

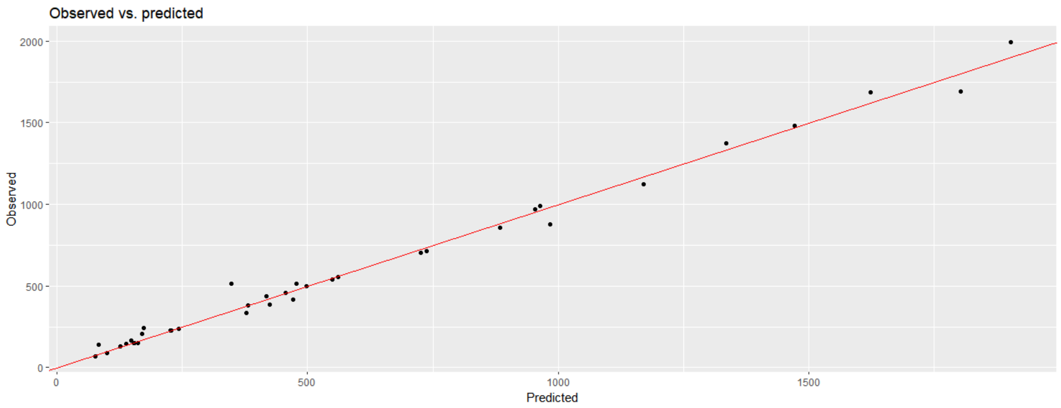

Figure S2. Observed vs. predicted values of the scale transformed by $h_{1/2}(\cdot)$ (see (2)). Residuals are the vertical distances between the identity line and observations. There is no apparent heterogeneous variance, e.g., no funnel-like cloud behavior, such as that which would be observed before transforming data using the square root transformation.

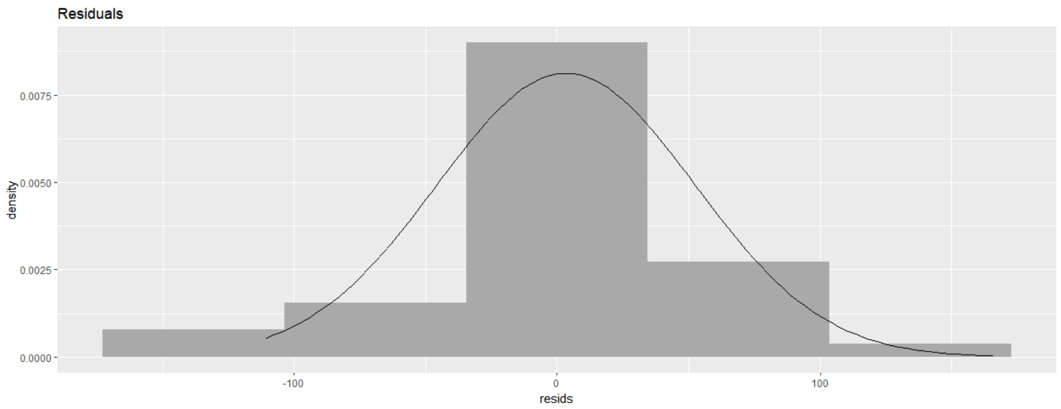

Figure S3. Histogram of residuals. There is no significant deviation from a normal distribution (Kolmogorov-Smirnov test statistic 0.13152, $p = 0.5027$).

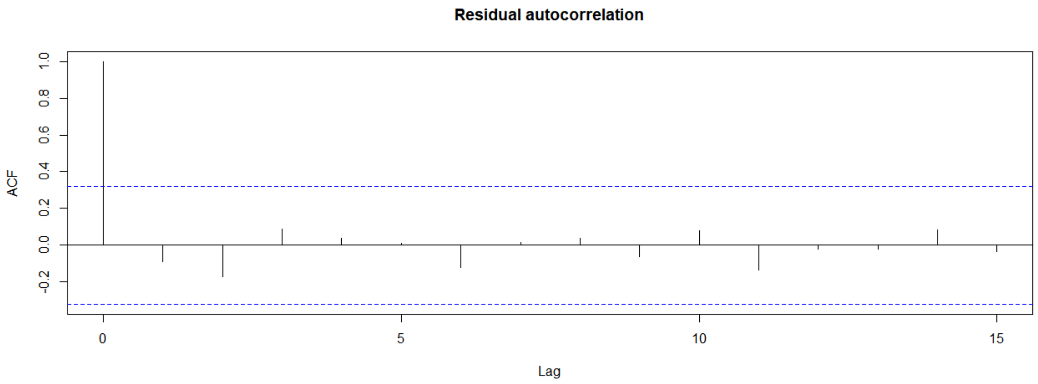

Figure S4. Autocorrelation plot of residuals. Based on a runs test, there is no significant autocorrelation (test statistic -0.85409, $p = 0.3931$).

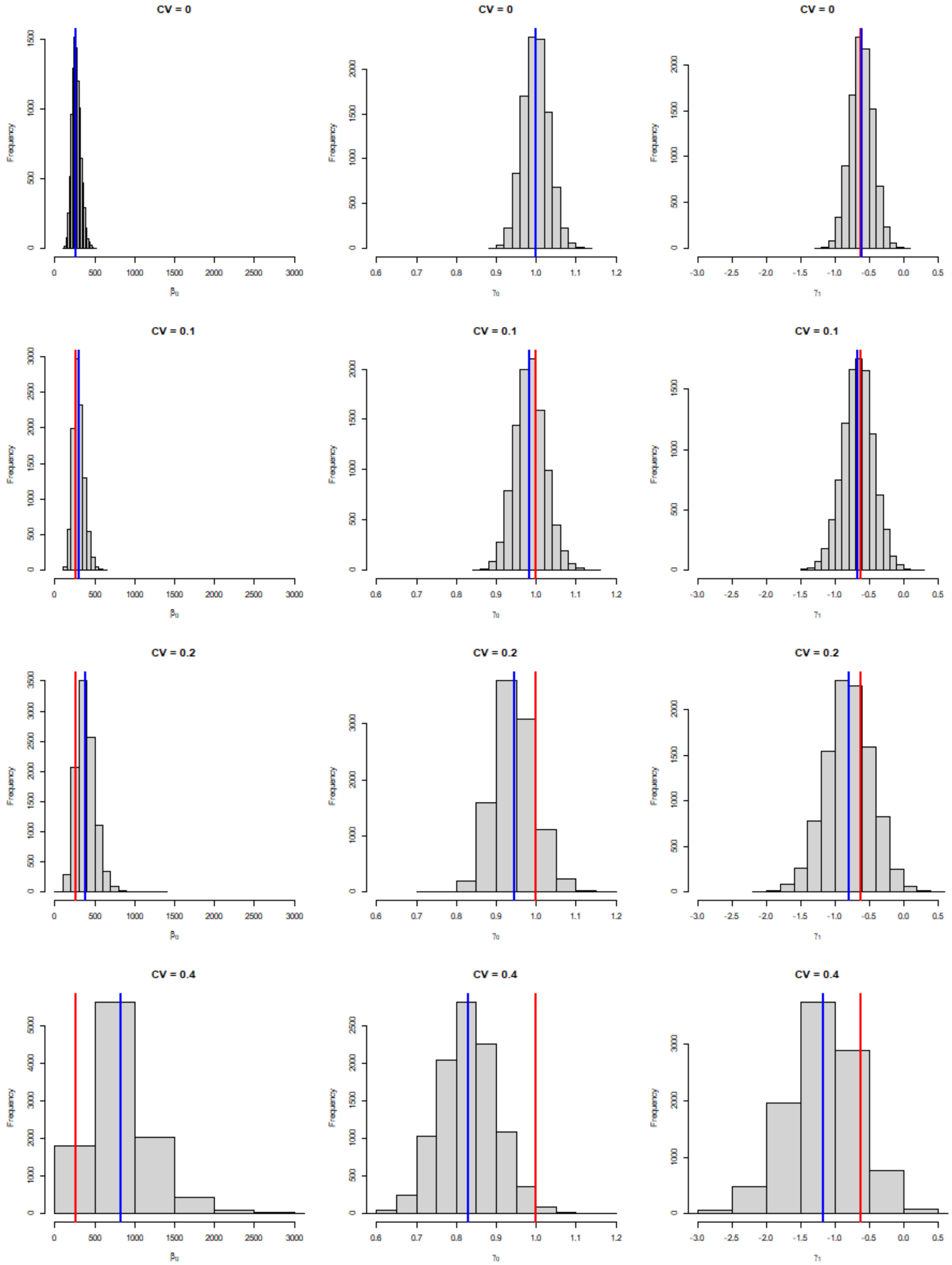

Figure S5. Sampling distributions of parameter estimates. The simulation study (10,000 replicates) with four different coefficients of variation for errors in the measurement of females involved the computation of the estimated parameters. The red line is located at the true value used for simulations and the blue line is the empirical mean of each sampling distribution.
